# Problematic cryptoasset trading is associated with greater depressive symptoms, anxiety symptoms, and social isolation

**DOI:** 10.1371/journal.pone.0349874

**Published:** 2026-05-26

**Authors:** Dar Meshi, Dayeoun Jang, Zijie Mei, Rabindra Ratan, Maxwell Foxman

**Affiliations:** 1 Department of Advertising and Public Relations, Michigan State University, East Lansing, Michigan, United States of America; 2 Department of Media and Information, Michigan State University, East Lansing, Michigan, United States of America; 3 School of Journalism and Communication, University of Oregon, Eugene, Oregon, United States of America; Beihang University, CHINA

## Abstract

Public blockchains allow individuals to own, trade, and transfer digital cryptoassets on the internet. These cryptoassets are highly volatile and available to trade 24 hours a day, seven days a week. The potential financial rewards stemming from buying and selling cryptoassets create the possibility for individuals to display a maladaptive behavior, problematic cryptoasset trading, which can be considered as a type of problematic gambling. Prior research has demonstrated that behavioral addictive disorders, like gambling disorder, are associated with poor mental health. In addition, previous research has revealed that individuals who simply trade cryptoassets describe experiencing more negative mental health symptoms in comparison to non-investing individuals. Therefore, we hypothesized that problematic cryptoasset trading would be positively associated with three measures of mental health: depressive symptoms, anxiety symptoms, and social isolation. We collected cross-sectional survey data (N = 239; female = 78, male = 161) and ran hierarchical linear regressions, controlling for demographic characteristics. Our analyses supported the hypothesized associations between problematic cryptoasset trading and the three mental health measures. In other words, higher levels of problematic cryptoasset trading were associated with higher levels of depressive symptoms, anxiety symptoms, and social isolation. Implications for further research and clinical practice are discussed.

## Introduction

Public blockchains are decentralized, geographically dispersed networks of computers that function to validate and record transactions into a shared, append-only digital ledger [1]. These blockchains can be considered public goods [2], and they provide an important, emergent service – they allow individuals to own, trade, and transfer digital cryptoassets on the internet. Cryptoassets are also referred to as “tokens,” and they are programmed to be either fungible (interchangeable, typically referred to as cryptocurrencies) or non-fungible (unique, typically referred to as NFTs, short for non-fungible tokens). For example, the native token of a blockchain (e.g., the “ether” token with the ticker ETH on the Ethereum blockchain), is a fungible token typically used to pay for transactions on the blockchain network. There are several other types of fungible tokens, such as tokens that are pegged to a government issued currency, termed stablecoins. On the other hand, NFTs are unique and consist of any type of computer file, so for example, an individual can own art (.jpg) or music (.wav) on a blockchain.

Although some of these cryptoassets are not intended to change in value (e.g., stablecoins), many are highly volatile, dramatically changing in value [3]. Therefore, individuals often buy them as investments, predicting that they will increase in value over time relative to their government’s currency [4,5]. In line with this, around 30% of Americans have owned at least one cryptoasset, and this number is increasing steadily, having doubled in the last three years [6,7]. People can buy cryptoassets using centralized exchanges (e.g., Coinbase), as well as decentralized exchanges (e.g., Uniswap). Importantly, unlike the traditional stock market, cryptoassets are available to buy and sell 24 hours a day, seven days a week, from anywhere in the world. This constant, year-round trading opportunity, combined with the highly volatile nature of these assets, creates the potential for problematic cryptoasset trading (PCT) [8,9]. In other words, individuals may develop a maladaptive relationship with cryptoasset trading due to the monetary rewards obtained through this behavior. A great deal of research has previously established that monetary rewards can drive maladaptive, problematic behavior in humans, such as with gambling disorder [10], as well as the problematic trading of stocks and other financial instruments [11]. Indeed, researchers have often considered the problematic trading of stocks as a subdomain of gambling disorder [11], and we conceptualize PCT in the same way.

Researchers have already warned of the potential for individuals to display PCT and theorized how PCT could possibly develop. For example, Delfabbro and colleagues [8] have proposed several risk factors for PCT, such as the illusion of control (subjective over-estimation of the objective ability to exert control over trades and obtain higher returns), social learning and reinforcement (observing others’ trading gains on social media), and preoccupation (a symptom of addictive behavior in which cryptoasset trading is constantly salient and top of mind). Furthermore, a digital context overlap exists between cryptoasset trading, gaming, and gambling, which appears to potentially exacerbate the degree to which individuals display PCT [12,13]. Indeed, initial qualitative research has demonstrated that cryptoasset traders find the trading experience similar to gambling, experience harms (e.g., distress) due to their trading, and enact strategies to reduce these harms [14,15]. For example, these individuals reported engaging in compulsive trading, displaying an obsession with tracking cryptocurrency prices throughout the day, neglecting work and personal relationships, damaging personal relationships due to financial losses, and needing to combat the urge to trade. Furthermore, several studies have demonstrated that cryptoasset trading is positively associated with the severity of excessive, problematic gambling [16–21], and cryptoasset trading has been associated with greater self-reported feelings of addiction [22]. Taken together, the nascent literature raises concerns regarding the potential for cryptoasset trading to develop into a problematic behavior. In response to this, calls have been made to further investigate the relationship between cryptoasset trading and key relevant variables and factors, such as mental health [9]. The relationship between problematic gambling and poor mental health has been well-documented [23], but the relationship between PCT and mental health remains underexplored.

To the best of our knowledge, only a handful of studies have investigated the relationship between cryptoasset trading and mental health constructs, such as depression, anxiety and social isolation [24,25]. Qualitative research has revealed that individuals who trade cryptoassets report related negative mental health, such as “depression after financial losses” and “anxiety due to market volatility” [15]. Regarding quantitative research on cryptoasset traders, one study revealed that cryptoasset trading frequency was positively associated with symptoms of depression and anxiety [16], while two other studies demonstrated that cryptoasset traders had greater trait anxiety [18] and loneliness [17] in comparison to non-investing individuals. Important to note, these quantitative studies primarily focused on whether individuals traded cryptoassets or not, and did not specifically assess the construct of PCT. In this regard, a single previous study has investigated the relationship between PCT and mental health [26]. The authors of this study adapted a measure of problematic social media use, replacing the term “social media” in the survey items with the term “cryptoassets,” and they also combined measures of depressive and anxiety symptoms into a single mental health construct. Their analysis, along with a host of other problematic behaviors, revealed a positive association between PCT and poorer mental health. This analysis, however, did not allow for the disentangling of depressive and anxiety symptoms, each of which is a separate diagnosis with different symptoms as outlined in the Diagnostic and Statistical Manual of Mental Disorders, Fifth Edition, Text Revision [27]. In addition, the authors did not assess loneliness or social isolation.

With the above in mind, the present study aims to better understand the relationship between PCT and distinct components of mental health. In line with the above-described literature, we hypothesized that higher levels of PCT would be associated with greater depressive symptoms, anxiety symptoms, and social isolation. To address our hypotheses, we conducted an online survey to collect measures of PCT and these mental health constructs. Of note, we took an approach similar to Ellithorpe and colleagues [26], using several of the same survey measures, however, we did not combine mental health constructs. Specifically, we conducted separate hierarchical linear regression analyses for each mental health construct to assess its relationship with PCT.

## Methods

### Ethics statement

This study was approved by the Michigan State University Institutional Review Board. All participants provided informed consent electronically prior to participation.

### Participants

Our final sample size was 239 participants (female = 78, 32.6%; male = 161, 67.4%), after excluding one participant for not fully completing the survey. The average age of our sample was 33.46 years (SD = 9.70; range = 18–68), and all participants reported living in the United States. Regarding race, 67 (28.0%) participants were White, 86 (36.0%) were Black, and 86 (36.0%) were Asian. We intentionally stratified our sample by these races during recruitment, and for the reported analyses, we dichotomized race into two categories (White = 67, 28.0%; and non-White = 172, 72.0%) to capture a commonly reported demographic pattern in cryptocurrency adoption, where racial minority groups in the United States are collectively more likely than White individuals to report owning or using cryptocurrencies [28]. Prior population surveys have documented higher adoption rates among Asian and Black adults compared with white adults. To ensure that this analytic decision did not influence our findings, we also conducted additional analyses using the three original racial categories (White, Black, Asian); these analyses produced substantively identical results.

Of note, the current sample was included in a previous study with a larger group of 604 participants, described in Lim *et al*. [29]. Importantly, however, participant inclusion into the current analysis required a response of “yes” to the following question “Do you own or invest in cryptoassets (e.g., cryptocurrencies, NFTs, etc.)?” with yes/no being the only response options. All the participants who answered “yes” also survived a “straight-line” response check with an extremely low standard deviation (less than 0.5) across all 5-point Likert scales in the surveys described below.

### Procedure

We recruited participants via Prolific, a high-quality online survey panel platform which has been found to offer engaged research participants [30]. All participants first provided informed, electronic consent and then voluntarily responded to the items in our survey. Upon conclusion of the survey, participants received $3.10, which if considering the average time of survey completion, was 70% higher than the federal minimum wage at the time of the recruitment. All data were collected in March of 2022 (data and author-generated analysis code are available at the Open Science Framework repository: https://osf.io/8xe4c/).

### Measures

#### Problematic cryptoasset trading.

To assess problematic cryptoasset trading, we adapted the 6-item Bergen Social Media Addiction Scale [31,32], as this specific scale adaptation was previously taken in Ellithorpe and colleagues [26]. In the adapted items, participants were asked about their investing in cryptoassets (e.g., cryptocurrencies, NFTs) over the past year. Thus, PCT in the present study refers to problematic engagement with cryptoasset investment behaviors. Participants were prompted with, “Please answer the following questions with regard to your investing in cryptoassets (e.g., cryptocurrencies, NFTs, etc.) over the past year” and each item assessed one of six commonly accepted core aspects of addiction [33]: preoccupation, mood modification, tolerance, conflict, withdrawal, and relapse. Responses were recorded on a five-point Likert scale (1 = very rarely; 5 = very often) and summed to create a total score ranging from 6 to 30, with greater scores indicating greater PCT. The full list of adapted items is provided in S1 Appendix. The internal consistency of the measure with our sample was good (Cronbach’s α = 0.87).

#### Depressive symptoms.

Depressive symptoms were measured using the 4-item short form of the PROMIS depression scale [34]. Participants were prompted with, “How often have you felt the following in the past 7 days?” and then asked four items about their mental state. For example, one item asked “I felt hopeless.” Participants responded to each item on a five-point Likert scale (1 = Never; 5 = Always). Responses were summed with a possible range of 4–20, with a greater score indicating greater depressive symptoms. The internal consistency of the measure with our sample was excellent (Cronbach’s α = 0.93).

#### Anxiety symptoms.

Anxiety symptoms were measured using the 4-item short form of the PROMIS anxiety scale [34]. Participants were prompted with, “How often have you felt the following in the past 7 days?” and then asked four items about their mental state. For example, one item asked “I felt fearful.” Participants responded to each item on a five-point Likert scale (1 = Never; 5 = Always). Responses were summed with a possible range of 4–20, with a greater score indicating greater anxiety symptoms. The internal consistency of the measure with our sample was excellent (Cronbach’s α = 0.92).

#### Social isolation.

Social isolation was measured using the 4-item short form of the PROMIS anxiety scale [35]. Participants were prompted with, “How often have you felt the following in the past 7 days?” and then asked four items about their feelings in regard to others. For example, one item asked “I felt left out.” Participants responded to each item on a five-point Likert scale (1 = Never; 5 = Always). Responses were summed with a possible range of 4–20, with a greater score indicating greater social isolation. The internal consistency of the measure with our sample was excellent (Cronbach’s α = 0.93).

#### Demographic characteristics.

Age, sex, and race were obtained from the Prolific platform (see above), and we also collected education and income level with a single item each in our survey. For education, we asked “What is your highest level of education attained?” and participants responded on an eight-point scale (1 = Some High-School Education; 8 = Completed doctoral education). The mean response of our sample was 4.08 (SD = 1.56) indicating “Completed undergraduate education.” For income level, we asked “What is your approximate yearly income?” and participants responded on an 11-point scale (1 = Less than $10,000; 11 = $100,000 or more). The mean response of our sample was 5.65 (SD = 3.40) indicating “$50,000 - $59,999.”

### Data analysis

Statistical analyses were performed using MATLAB (version R2022B). We first conducted exploratory correlation analyses, computing Pearson’s correlation coefficients between all continuous variables, point-biserial correlations between continuous variables and dichotomous variables, and Pearson’s chi-square-based phi correlation when both variables were dichotomous. To directly address our hypotheses, we conducted hierarchical linear regression analyses with PCT as the independent variable. Each model included one dependent variable representing a mental health measure: depressive symptoms, anxiety symptoms, or social isolation. Demographic variables (age, sex, race, education, and income) were entered in Block 1 as control variables, and PCT was entered in Block 2 to examine whether it accounted for additional variance in the mental health measures beyond demographic characteristics. There were no significant violations of the assumptions of homoscedasticity, independence of errors, and normality of residuals in all models according to the Breusch-Pagan test, Durbin-Watson test, and Shapiro-Wilk test. Variable inflation factors were calculated to assess multicollinearity among the independent variables, and there was no evidence of multicollinearity. We also performed Bonferroni correction for multiple comparisons on our findings.

## Results

We first conducted exploratory correlation analyses with all variables. Please see Table 1 for these correlation statistics, as well as means and standard deviations of variables. These analyses revealed significant positive associations between problematic cryptoasset trading and each of our three measures of mental health: depressive symptoms (*r* = .18, *p* = .005), anxiety symptoms (*r* = .22, *p* < .001), and social isolation (*r* = .15, *p* = .022). In addition, problematic cryptoasset trading was positively correlated with sex (*r* = .15, *p* = .019) and negatively correlated with race (*r* = −.17, *p* = .007). Due to our variable coding, this revealed that males and non-white individuals were more likely to have a greater degree of problematic cryptoasset trading.

**Table 1:** Central tendency and Pearson’s pairwise bivariate correlation matrix of all model variables (*N* = 239).

	1.	2.	3.	4.	5.	6.	7.	8.	9.
Mean	10.82	7.91	8.06	8.45	33.46	–	–	4.08	5.65
SD	4.64	4.00	3.82	4.26	9.70	–	–	1.56	3.40
1. PCT	–	.18**	.22***	.15*	.00	.15*	−.17**	.03	.10
2. Depressive Symptoms		–	.85***	.67***	−.09	−.10	.07	−.21***	−.23***
3. Anxiety Symptoms			–	.68***	−.09	−.12	.11	−.22***	−.24***
4. Social Isolation				–	−.02	−.07	.02	−.13*	−.19**
5. Age					–	−.01	.11	.30***	.14*
6. Sex^a^						–	.00	−.03	.17**
7. Race^b^							–	.00	−.12
8. Education^c^								–	.42***
9. Income^d^									–

Note: PCT = Problematic Cryptoasset Trading. Correlations of continuous variables with the dichotomous sex and race variables are point-biserial correlations, and the correlation between the dichotomous sex and race variables is a Pearson’s chi-square-based phi correlation. * *p* < .05, ** *p* < .01, *** *p* < .001.

^a^Sex was coded as Female = 0 and Male = 1. Mean and SD omitted, please see Methods for sample characteristics.

^b^Race was coded as non-White = 0 and White = 1. Mean and SD omitted, please see Methods for sample characteristics.

^c^Mean number represents the nearest category, specifically, 4.08 represents “Completed undergraduate education.”

^d^Mean number represents the nearest category, specifically, 5.65 represents “$50,000 - $59,999.”

We next addressed our hypotheses by conducting three separate hierarchical linear regression analyses (Table 2). In these analyses, demographic variables (age, sex, race, education, and income) were entered in Block 1, and PCT was entered in Block 2. In this way, we examined whether problematic cryptoasset trading was associated with each of our three measures of mental health beyond demographic characteristics. Our analyses revealed that problematic cryptoasset trading was positively associated with depressive symptoms (β = .23, p < .001), anxiety symptoms (β = .29, p < .001), and social isolation (β = .18, p < .01). In addition, problematic cryptoasset trading explains additional variance beyond demographic factors in each model (ΔR^2^ = .03–.08). In other words, higher levels of problematic cryptoasset trading were associated with poorer mental health.

**Table 2:** Hierarchical linear regressions with demographic characteristics and problematic cryptoasset trading predicting mental health measures (*N* = 239)*.*

Variable	Model 1 Depressive Symptoms			Model 2 Anxiety Symptoms			Model 3 Social Isolation		
	*β*	SE	95% CI	*β*	SE	95% CI	*β*	SE	95% CI
Block 1									
Age	−.04	.06	[-.16,.09]	−.04	.06	[-.16,.09]	.02	.07	[-.11,.15]
Sex^a^	−.11	.06	[-.23,.02]	−.14^*^	.06	[-.26, -.02]	−.08	.07	[-.20,.05]
Race^b^	.09	.06	[-.03,.22]	.14^*^	.06	[.02,.26]	.03	.07	[-.10,.16]
Education	−.14^*^	.07	[-.28, -.01]	−.15^*^	.07	[-.29, -.01]	−.08	.07	[-.22,.06]
Income	−.16^*^	.07	[-.30, -.02]	−.15^*^	.07	[-,29, -.03]	−.16^*^	.07	[-.30, -.02]
*R* ^*2*^	.080^**^			.091^***^			.042		
Block 2									
PCT	.23^***^	.06	[.11,.36]	.29^***^	.06	[.17,.41]	.18^**^	.07	[.06,.31]
*R* ^*2*^	.131^***^			.170^***^			.074^**^		
*ΔR* ^*2*^	.052^***^			.079^***^			.031^**^		

Note: PCT = Problematic Cryptoasset Trading. Coefficients are standardized. *p < .05, **p < .01, ***p < .001.

^a^Sex was coded as Female = 0 and Male = 1 before standardization.

^b^Race was coded as non-White = 0 and White = 1 before standardization.

We also conducted additional regression analyses to examine whether the association between PCT and our mental health measures differed across demographic characteristics. Specifically, we added interaction terms between PCT and age, sex, race, education, and income into Block 2 of our hierarchical regression models. None of these interaction terms were statistically significant for depressive symptoms, anxiety symptoms, or social isolation (all p’s > .05). This suggests that the observed associations between PCT and our mental health measures did not differ significantly across these demographic characteristics in the present sample. The full results of these moderation analyses are provided in S1 Table.

## Discussion

In the present study, we collected cross-sectional survey data and ran three hierarchical linear regressions with PCT as the independent variable, controlling for age, sex, race, education, and income. Each model predicted a different mental health measure: depressive symptoms, anxiety symptoms, or social isolation. Our results revealed significant positive associations between PCT and all three of our measures of mental health. To the best of our knowledge, only one other study has assessed problematic cryptoasset investing and related this construct to a measure of mental health [26]. Our results align with and extend these findings, demonstrating a similar directional relationship, but distinctly with depressive and anxiety symptoms, and with a mental health measure that had not been previously assessed, social isolation. Our results also align with research on other problematic behaviors, such as gambling disorder [23], problematic social media use [36], and problematic gaming [37] – the greater degree to which individuals experience these problematic behaviors, the poorer their mental health.

Regarding our analyses, the proportion of variance explained by the regression models was modest (R² = .07–.17). However, such values are typical in psychological survey research, particularly when examining complex mental health constructs that reflect many biological, psychological, and social influences. Importantly, problematic cryptoasset trading explained additional variance beyond demographic factors in all three models (ΔR² = .03–.08), indicating that PCT accounted for a small but meaningful proportion of variance in depressive symptoms, anxiety symptoms, and social isolation.

Additional moderation analyses did not provide evidence that the associations between problematic cryptoasset trading and the mental health measures differed significantly by age, sex, race, education, or income. This suggests that the observed relationships between PCT and depressive symptoms, anxiety symptoms, and social isolation were relatively consistent across demographic groups in the present sample. However, these null findings should be interpreted cautiously given the sample size and the typically small magnitude of interaction effects in psychological survey research.

Although we examined the above-described moderators, we did not directly examine the causal mechanisms underlying our revealed relationships. Therefore, we can only speculate about what could be driving the relationship between PCT and poor mental health. Insights from research on related problematic behaviors, such as gambling disorder, may offer useful guidance, as researchers in those areas have already investigated relationships to mental health (for review see [10]). For example, it could be that PCT shares underlying neurobiological or psychological vulnerabilities with mental health conditions. Alternately, negative outcomes from PCT, such as financial losses or stress related to market volatility, may contribute to depressive symptoms, anxiety symptoms, or social isolation. Future research on PCT can address these putative mechanisms more directly, for example using longitudinal or experimental designs, to better understand whether the association between PCT and mental health reflects a unidirectional causal relationship, a bidirectional relationship, or third-variable explanations, such as shared risk factors.

Despite the insights provided by the current research, our study has limitations that we would like to mention. First, the data are cross-sectional in nature, so we cannot determine a causal relationship between our variables. In other words, it could be that PCT leads to poor mental health, or it could be that poor mental health leads to PCT. As mentioned above, we look forward to future research that can address the nature of this relationship. In addition, our measures of mental health were brief, four-item scales. Although these scales have been validated [34,35] and we utilized these scales to avoid participant burden, it could be that these scales potentially lack a thorough, comprehensive assessment of these constructs. Future researchers can implement longer, more detailed scales to replicate our findings. Finally, we adapted the Bergen Social Media Addiction Scale to measure our construct of interest, PCT. Although the Bergen Social Media Addiction Scale has been validated [31,32] and our specific adaptation has been previously employed [26], it is important to note that this adapted scale has not been validated. Therefore, our findings should be interpreted with caution.

In sum, we found that higher levels of problematic cryptoasset trading were associated with higher levels of depressive symptoms, anxiety symptoms, and social isolation. The present study provides clinicians with current, relevant information about the relationship between PCT and mental health. As the prevalence of cryptoasset trading continues to grow [6], clinicians should be aware that individuals reporting problematic engagement with cryptoasset trading may also report poorer mental health. These findings may help inform clinical assessment and future research on interventions, although more research is needed to determine the exact nature of the relationship between PCT and mental health.

## Supporting information

S1 Appendix Adapted Problematic Cryptoasset Investing Scale. (DOCX)

S1 Table Hierarchical linear regressions with demographic characteristics and problematic cryptoasset trading predicting mental health measures (*N* = 239). (DOCX)
