# Supplementary material for: Problematic cryptoasset trading is associated with greater depressive symptoms, anxiety symptoms, and social isolation
Source: PLoS One. 2026 May 26;21(5):e0349874. doi: 10.1371/journal.pone.0349874 (PMC13210369; doi:10.1371/journal.pone.0349874)
Supplement: S1 Appendix — (DOCX) [file pone.0349874.s001.docx]

**S1 Appendix. Adapted Problematic Cryptoasset Investing Scale.**

Prompt:

Please answer the following questions with regard to your investing in cryptoassets (e.g., cryptocurrencies, NFTs, etc.). How often during the last year have you…

Response scale:

1 = Very rarely

2 = Rarely

3 = Sometimes

4 = Often

5 = Very often

Items:

1. Spent a lot of time thinking about investing or plan how you will invest in cryptoassets?

2. Felt an urge to invest more and more in cryptoassets?

3. Invested in cryptoassets in order to forget about personal problems?

4. Tried to cut down on your investing in cryptoassets without success?

5. Become restless or troubled if you are prohibited from investing in cryptoassets?

6. Invested in cryptoassets so much that it had a negative impact on your job/studies/hobbies?

Items adapted from:

Andreassen CS, Torsheim T, Brunborg GS, Pallesen S. Development of a Facebook Addiction Scale. Psychol Rep. 2012;110: 501–517. Available: http://www.ncbi.nlm.nih.gov/pubmed/22662404

Bányai F, Zsila Á, Király O, Maraz A, Elekes Z, Griffiths MD, et al. Problematic social media use: Results from a large-scale nationally representative adolescent sample. PLoS One. 2017;12: e0169839. doi:10.1371/journal.pone.0169839
