# Supplementary material for: Problematic cryptoasset trading is associated with greater depressive symptoms, anxiety symptoms, and social isolation
Source: PLoS One. 2026 May 26;21(5):e0349874. doi: 10.1371/journal.pone.0349874 (PMC13210369; doi:10.1371/journal.pone.0349874)
Supplement: S1 Table — (DOCX) [file pone.0349874.s002.docx]

**S1 Table. Hierarchical linear regressions with demographic characteristics and problematic cryptoasset trading predicting mental health measures (*N* = 239)*.***

| Variable | Model 1  Depressive Symptoms | | | Model 2  Anxiety Symptoms | | | Model 3  Social Isolation | | |
| --- | --- | --- | --- | --- | --- | --- | --- | --- | --- |
|  | *β* | SE | 95% CI | *β* | SE | 95% CI | *β* | SE | 95% CI |
| Block 1 |  |  |  |  |  |  |  |  |  |
| Age | -.03 | .07 | [-.16, .10] | -.04 | .06 | [-.16, .09] | .04 | .07 | [-.09, .17] |
| Sex^a^ | -.11 | .07 | [-.24, .02] | -.14^*^ | .06 | [-.27, -.02] | -.07 | .07 | [-.20, .06] |
| Race^b^ | .09 | .07 | [-.04, .21] | .14^*^ | .06 | [.02, .27] | .03 | .07 | [-.10, .16] |
| Education | -.16^*^ | .07 | [-.30, -.02] | -.15^*^ | .07 | [-.29, -.02] | -.09 | .07 | [-.23, .05] |
| Income | -.17^*^ | .07 | [-.31, -.03] | -.16^*^ | .07 | [-,29, -.02] | -.16^*^ | .07 | [-.30, -.02] |
| *R^2^* | .080^**^ |  |  | .091^***^ |  |  | .042 |  |  |
| Block 2 |  |  |  |  |  |  |  |  |  |
| PCT | .24^***^ | .06 | [.11, .36] | .29^***^ | .06 | [.17, .42] | .18^**^ | .07 | [.05, .30] |
| PCT×Age | -.10 | .06 | [-.22, .03] | -.00 | .06 | [-.12, .12] | -.08 | .07 | [-.21, .05] |
| PCT×Sex | .03 | .07 | [-.11, .17] | .01 | .07 | [-.12, .15] | .10 | .07 | [-.04, .24] |
| PCT×Race | .00 | .07 | [-.13, .14] | .02 | .07 | [-.12, .15] | .08 | .07 | [-.06, .22] |
| PCT×Education | .11 | .07 | [-.03, .25] | .06 | .07 | [-.07, .20] | .01 | .07 | [-.13, .15] |
| PCT×Income | -.08 | .07 | [-.22, .06] | -.06 | .07 | [-.20, .08] | .05 | .07 | [-.10, .20] |
| *R^2^* | .149^***^ |  |  | .175^***^ |  |  | .104^**^ |  |  |
| *ΔR^2^* | .069^**^ |  |  | .084^**^ |  |  | .062^*^ |  |  |

Note: PCT = Problematic Cryptoasset Trading. Coefficients are standardized. **p* < .05, ***p* < .01, ****p* < .001.

^a^ Sex was coded as Female=0 and Male=1 before standardization.

^b^ Race was coded as non-White=0 and White=1 before standardization.
